# Ofloxacin-resistant Vibrio cholerae O139 in Hong Kong.

**DOI:** 10.3201/eid0504.990431

**Published:** 1999

**Authors:** K M Kam, K Y Luey, T L Cheung, K Y Ho, K H Mak, P T Saw


These are the first reported human cases of West Nile fever in Central Europe (5); an extensive outbreak occurred in Romania in 1996, with approximately 500 patients hospitalized and a 4% to 8% fatality rate (6,7). West Nile virus should be viewed as a potential agent of local sporadic cases, clusters, or outbreaks, even in temperate Europe. Environmental factors (including human activities) that enhance vector population densities (heavy rains followed by floods, irrigation, higher than usual temperatures due to global warming) might produce an increased incidence of West Nile fever and other new or reemerging mosquito-borne diseases. Surveillance for West Nile fever should monitor population density and infection rate of principal vectors, antibodies in vertebrates and exposed human groups, and routine diagnosis of human infections.

**Zdenek Hubálek, Jirí Halouzka, and Zina Juricová**
Institute of Vertebrate Biology, Academy of Sciences, Brno, Czech Republic

### References

1. Hubálek Z, Halouzka J, Juricová Z, Šebesta O. First isolation of mosquito-borne West-Nile virus in the Czech Republic. Acta Virol 1998;42:119-20.
2. Melnick JL, Paul JR, Riordan JF, Barnett VHH, Goldblum N, Zabin E. Isolation from human sera in Egypt of a virus apparently identical to West Nile virus. Proc Soc Exp Biol Med 1951;77:661-5.
3. de Madrid AT, Porterfield JS. The flaviviruses (group B arboviruses): a cross-neutralization study. J Gen Virol 1974;23:91-6.
4. Leake CJ, Varma MGR, Pudney M. Cytopathic effect and plaque formation by arboviruses in a continuous cell line (XTC-2) from the toad *Xenopus laevis*. J Gen Virol 1977;35:335-9.
5. Hubálek Z, Halouzka J. Arthropod-borne viruses of vertebrates in Europe. Acta Scientiarum Naturalium Academiae Scientiarum Bohemicae Brno 1996;30, no. 4-5:1-95.
6. Le Guenno B, Bougermouh A, Azzam T, Bouakaz R. West Nile: a deadly virus? Lancet 1996;348:1315.
7. Tsai TF, Popovici F, Cernescu C, Campbell GL, Nedelcu NI. West Nile encephalitis epidemic in southeastern Romania. Lancet 1998;352:767-71.

### Ofloxacin-Resistant *Vibrio cholerae* O139 in Hong Kong

**To the Editor:** Unexpected outbreaks of cholera occurred in many areas of the world in 1997-98, partly because of weather changes associated with the El Niño phenomenon (1). Outbreaks caused by antibiotic-resistant *Vibrio cholerae* O1 and O139 have been documented in the Indian subcontinent (2-4), Africa (5), and Ukraine (6).

In Hong Kong, nonduplicate bacterial strains of *V. cholerae* O1 and O139 isolated from patients and environmental sources and received in the Public Health Laboratory between January 1, 1993, and June 30, 1998, were identified by conventional biochemical tests (7,8) and API 20E (bioMerieux, France); serotyped by slide agglutination with polyvalent O1 and mono-specific Inaba and Ogawa antisera (Murex, Dartford, United Kingdom); and checked with O139 antiserum (Denka Seiken, Tokyo, Japan). Biotyped and antibiotic susceptibilities were determined by the Kirby-Bauer disk-diffusion assay (8-10). Antibiotics tested included chloramphenicol and tetracycline (from 1993 to 1996) and ofloxacin (added in routine testing from 1997). *V. cholerae* isolates available for further study were tested with the standard broth microdilution method (11) to measure minimum inhibitory concentrations (MICs) of susceptibilities to chloramphenicol, tetracycline, and ofloxacin.

No antibiotic resistance was seen in *V. cholerae* isolates in testing conducted from 1969 to 1995. The first *V. cholerae* isolate with reduced susceptibility to chloramphenicol but sensitive to tetracycline was encountered in Hong Kong in 1996. This O1 El Tor Ogawa strain was imported from Nepal. Since then, more O1 strains were isolated that exhibited reduced antibiotic susceptibilities to chloramphenicol and tetracycline but not to ofloxacin (12). In May 1998, seven *V. cholerae* O139 strains were isolated that displayed patterns of antibiotic susceptibilities strikingly different from those of O1 isolates; the former were all sensitive to tetracycline but showed reduced susceptibilities to chloramphenicol and ofloxacin. All *V. cholerae* O1 strains tested have been susceptible to ofloxacin; O1 isolates falling into intermediate categories for chloramphenicol and tetracycline susceptibilities (31% and 27.6%, respectively) were common.

The first isolate of *V. cholerae* O139 in Hong Kong came from the imported case of a patient who had traveled to other provinces of China (13,14). Isolation of O139 continued sporadically since then, with six cases between 1993 and the

1st quarter of 1998. In May 1998, a cluster of seven imported cases of *V. cholerae* O139 were reported with strains isolated from seven persons who became ill with severe diarrhea after visiting Zhuhai in Guangdong Province, China. Of 13 *V. cholerae* O139 isolates tested, 7 showed intermediate resistance to chloramphenicol and high-level resistance to ofloxacin (MIC 16 µg/ml) but no resistance to tetracycline (MIC 50s and MIC 90s were 0.25 µg/ml). This is the first evidence of a quinolone-resistant strain of *V. cholerae* O139 in Hong Kong. Of the O1 isolates, none were resistant to chloramphenicol and ofloxacin, but six were resistant to tetracycline (MIC 50s and MIC 90s were 0.25 µg/ml and 8 µg/ml, respectively).

Although all O1 isolates were sensitive to chloramphenicol, there was only a twofold difference in MIC90 to chloramphenicol between O1 and O139 isolates. MIC90s of ofloxacin for O139 were nearly 10 times higher than those for O1 strains.

The novel appearance of O139 resistant to ofloxacin with MICs of 16 µg/ml from Guangdong Province, China, was of special concern. Preliminary results using pulsed-field gel electrophoresis analysis of chromosomal DNA showed that these ofloxacin-resistant O139 strains had identical fingerprint patterns and probably belonged to the clone that had caused severe diarrheal disease in the region. Two previous surveys of *V. cholerae* antibiotic susceptibilities had not described any ofloxacin-resistant O139 strains (15,16). The potential for rapid spread of these strains threatens cholera prevention and control efforts that may still rely on chemotherapy.

Different antimicrobial resistance patterns of *V. cholerae* O1 and O139 were noted. Among the resistant O1 isolates, four were local, one was from other provinces of China, and one was from Thailand. All the resistant O139 isolates were imported from Guangdong Province, China. Antibiotic resistance was found in strains from local isolates and from neighboring countries. The unique patterns of antimicrobial resistance for the O1 and O139 isolates suggest different mechanisms of resistance. As quinolones are used heavily in this region to treat cholera and other enteric diseases, selective pressure could encourage emergence of ofloxacin resistance. Prudent use of antibiotics should be exercised during antimicrobial therapy and prophylaxis for cholera and other enteric diseases to decrease the selection of more resistant clones in our locality.

**Kai Man Kam, Kit Yee Luey, Tze Leung Cheung, Kwan Yee Ho, Kwok Hang Mak, and Paul Thian Aun Saw**
Department of Health, Hong Kong SAR Government

### References

1. World Health Organization, Geneva. Cholera in 1997. Wkly Epidemiol Rec 1998;73:201-8.
2. Gharagozloo RA, Naficy K, Mouin M, Nassirzadeh MH, Yalda R. Comparative trial of tetracycline, chloramphenicol, and trimethoprim/sulphamethoxazole in eradication of *Vibrio cholerae* El Tor. BMJ 1970;4:281-2.
3. Glass RI, Huq I, Alim AR, Yunus M. Emergence of multiply antibiotic-resistant *Vibrio cholerae* in Bangladesh. J Infect Dis 1980;142:939-42.
4. Ramamurthy T, Garg S, Sharma R, Bhattacharya SK, Nair GB, Shimada T, et al. Emergence of novel strain of *Vibrio cholerae* with epidemic potential in southern and eastern India. Lancet 1993;341:703-4.
5. Finch MJ, Morris JG Jr, Kaviti J, Kagwanja W, Levine MM. Epidemiology of antimicrobial resistant cholera in Kenya and east Africa. Am J Trop Med Hyg 1988;39:484-90.
6. Clark CG, Kravetz AN, Alekseenko VV, Krendelev YuD, Johnson WM. Microbiological and epidemiological investigation of cholera epidemic in Ukraine during 1994 and 1995. Epidemiol Infect 1998;121:1-13.
7. Cowan ST, Steel KJ. Manual for the identification of medical bacteria. 2nd ed. Cambridge: Cambridge University Press; 1974.
8. Bradfold AK, Bopp CA, Wells JG. Isolation and identification of *Vibrio cholerae* O1 from fecal specimens. In: Wachsmuth IK, Blake AB, Olsvik Ø, editors. *Vibrio cholerae* and cholera: molecular to global perspectives. Washington: American Society for Microbiology; 1994. p. 3-26.
9. National Committee for Clinical Laboratory Standards. Performance standards for antimicrobial disk susceptibility tests approved standard. NCCLS document M2-A6. Villanova (PA): The Committee; 1997.
10. National Committee for Clinical Laboratory Standards. Performance standards for antimicrobial susceptibility testing; NCCLS document M100-S8, 18(1). Villanova (PA): The Committee; 8th information supplement, 1998.
11. National Committee for Clinical Laboratory Standards. Methods for dilution antimicrobial susceptibility tests for bacteria that grow aerobically. 4th ed. NCCLS document M7-A4. Villanova (PA): The Committee; 1997.
12. Wong W, Ho YY. Imported cholera cases among tours returning from Thailand. Public Health & Epidemiology Bulletin, Department of Health, Hong Kong. 1998;7:21-4.
13. Kam KM, Leung TH, Ho PYY, Ho KY, Saw TA. Outbreak of *Vibrio cholerae* O1 in Hong Kong related to contaminated fish tank water. Public Health 1995;109:389-95.

14. Lee SH, Lai ST, Lai JY, Leung NK. Resurgence of cholera in Hong Kong. Epidemiol Infect 1996;117:43-9.
15. Yamamoto T, Nair GB, Albert MJ, Parodi CC, Takeda Y. Survey of in vitro susceptibilities of *Vibrio cholerae* O1 and O139 to antimicrobial agents. Antimicrob Agents Chemother 1995;39:241-4.
16. Sciortino CV, Johnson JA, Hamad A. Vitek system antimicrobial susceptibility testing of O1, O139, and non-O1 *Vibrio cholerae.* J Clin Microbiol 1996;34:897-900.

### Plant Pathology and Public Health

> The day will come when the sign of the plant pathologist will stand forth in the street alongside that of the physician and surgeon. . . . For what will it profit us if all the ills and diseases of the human race be banished and we then face starvation because of diseases and pests in our food (1).

**To the Editor:** Every year plant diseases affect human society, resulting in inadequate nutrition and economic loss. The potato famine in the mid-1800s is the best-known example of a fungal plant pathogen's effect on history (2-4); *Phytopthora infestans* has recently reemerged in the Americas (5). Among the silent problems that have enormous effects on human society each year are crop infections by geminiviruses and tomato spotted wilt virus (6). These plant viruses are transmitted by whiteflies, leafhoppers, or thrips to hundreds of species of plants. They cause diseases of crops and ornamental plants around the world.

More obvious problems include ergotism, caused by the alkaloids produced by the fungus *Claviceps purpurea*. Ergotism was associated with the growth of rye, particularly in cool climates that cannot support wheat, and was implicated in the aberrant human behavior responsible at least in part for the Salem witch trials and St. Anthony's fire (2,7). In the last 5 years, a new plant disease, sorghum ergot (*Claviceps africana*), has spread north from Brazil into the United States. This fungus also causes disease in Australia, a sudden change from its known occurrence in Africa (8). Sorghum is the fifth most important cereal crop in the world, with approximately 45 million hectares under cultivation for food, beverages, feed, and fodder (8). Ergot alkaloid toxicity has not yet been demonstrated, but potential nutritional and economic losses could have substantial impact on public health.

With our increased awareness of the fragility of the environment, including the quality of our drinking water, opportunities may exist for physicians to interact with plant pathologists. Concern is growing about the use of *Burkholderia cepacia*, a bacterial phytopathogen, for the biologic control of seedling diseases (9). Although *B. cepacia* is effective for the biologic control of fungal diseases in the agricultural environment (10), this bacterium could contaminate the public water supply and subsequently influence the health of the immunosuppressed or persons with cystic fibrosis (9-11). This risk exemplifies the need to integrate plant health measures with human and veterinary health guidelines.

Plant pathology and public health also intersect with post-harvest fungal infections of seed and grain, particularly *Aspergillus flavus* and *Fusarium moniliforme* (2), which produce aflatoxin and fumonisin, respectively. During the past 2 drought years in Texas, aflatoxin in contaminated corn and peanuts has become a public health problem. In 1998, more than 50 pet dogs died of aflatoxicosis, perhaps by eating aflatoxin B1-contaminated corn used in dog food (12).

Although the veterinary and medical communities are well aware of the risks associated with plant pathogens when they enter the animal or human food supply, more routine interactions with plant pathologists could benefit public health. For example, plant pathologists can often predict impending plant disease outbreaks. This information can be used by epidemiologists to sound a warning about impending food shortages or poor food quality, particularly in developing countries. Plant pathologists are also developing new types of resistance in host plants and alternative strategies for managing plant diseases. These measures should improve food quality and reduce the negative public health impact associated with plant diseases.

**Karen-Beth G. Scholthof**
Texas A&M University, College Station, Texas, USA

### References

1. Whetzel HH. The relation of plant pathology to human affairs. Mayo Foundation Lectures 1926-1927. Philadelphia: W.B. Saunders Co.; 1928. p. 151-78.
2. Hudler GW. Magical mushrooms, mischievous molds. Princeton: Princeton University Press; 1998.
3. Woodham-Smith C. The great hunger: Ireland 1845-1849. New York: Old Town Books; 1962.

